# Detection of tuberculosis in cynomolgus macaques (*Macaca fascicularis*) using a supplementary Monkey Interferon Gamma Releasing Assay (mIGRA)

**DOI:** 10.1038/s41598-020-73655-3

**Published:** 2020-10-07

**Authors:** S. Warit, P. Billamas, N. Makhao, S. Jaitrong, T. Juthayothin, W. Yindeeyoungyeon, K. Dokladda, N. Smittipat, T. Kemthong, S. Meesawat, N. Kongsombat, C. Kraitat, T. Prammananan, T. Palaga, A. Chaiprasert, S. Malaivijitnond

**Affiliations:** 1 Industrial Tuberculosis Team, Industrial Medical Molecular Biotechnology Research Group, National Center for Genetic Engineering and Biotechnology, National Science and Technology Development Agency, 113 Thailand Science Park, Phahonyothin Road, Khlong Nueng, Khlong Luang, 12120 Pathum Thani Thailand; 2 National Primate Research Center of Thailand-Chulalongkorn University, Saraburi, 18110 Thailand; 3 Department of Biology, Faculty of Science, Chulalongkorn University, Bangkok, 10330 Thailand; 4 Department of Microbiology, Faculty of Science, Chulalongkorn University, Bangkok, 10330 Thailand; 5 Office for Research and Development, Faculty of Medicine Siriraj Hospital, Mahidol University, Bangkok, 10700 Thailand

**Keywords:** Microbiology, Infectious-disease diagnostics

## Abstract

Cynomolgus monkeys (*Macaca fascicularis*; MF) are commonly used as nonhuman primate models for pharmaceutical product testing. In their habitat range, monkeys have close contact with humans, allowing the possibility of bidirectional transmission of tuberculosis (TB) between the two species. Although the intradermal tuberculin skin test (TST) is used for TB detection in MF, it has limitations. Herein, we established the mIGRA, combining human QuantiFERON-TB Gold-Plus and monkey IFN-γ ELISA^pro^ systems, and used it to investigate 39 captive MF who were cage-mates or lived in cages located near a monkey who died from the naturally TB infection. During a 12-month period of study, 14 (36%), 10 (26%), and 8 (21%) monkeys showed TB-positive results using the mIGRA, the TST, and TB culture, respectively. Among the 14 mIGRA-positive monkeys, 8 (57.1%) were TST-positive and 7 (50%) were culture-positive, indicating early TB detection in the latent and active TB stages with the mIGRA. Interestingly, 3 (37.5%) of the TST-negative monkeys were culture-positive. Our study showed that the mIGRA offers many advantages, including high sensitivity and high throughput, and it requires only one on-site visit to the animals. The assay may be used as a supplementary tool for TB screening in MF.

## Introduction

Tuberculosis (TB) is a chronic airborne disease that causes high morbidity and mortality in both humans and nonhuman primates (NHPs), especially captive macaque monkeys. In NHPs, *Mycobacterium tuberculosis (M. tb)* is the main causative agent, while other members of *M. tb* complex (MTBC), such as *Mycobacterium bovis* (*M. bovis*) and nontuberculous mycobacteria (NTM), such as *Mycobacterium kansasii* (*M. kansasii*)*,* are associated with TB-like diseases^1–4^. *M. tb* infection in NHPs mostly occurs by close contact with TB-infected humans through the inhalation of aerosolized bacteria or other routes, such as ingestion, wounds, or animal tattooing^5,6^.

In experimental TB research, rhesus (*Macaca mulatta*) and cynomolgus (*Macaca fascicularis*) macaques are NHPs that have been widely used for basic studies on immunology and pathogenesis as well as vaccine and drug development because both species exhibit a full spectrum of human TB pathology (latent, subclinical, and active)^7–10^. However, several factors need to be considered prior to the selection and use of animals for TB research, such as the geographic source of macaques, *M. tb* strain used (e.g., H37R*v*, CDC1551, or Erdmann), and route and dose of infection^7–9,11,12^. Infection with an extremely low dose (approximately 25 colony forming units) of the *M. tb* Erdman strain in Asian cynomolgus macaques demonstrated that these macaques could remain subclinically and/or latently infected with TB for a long period (14–20 months), similar to humans^7–9,11^. Moreover, the study of the reactivation of latent TB infection in cynomolgus macaques could be performed through the use of anti-tumor necrosis factor (anti-TNF) antibodies^13^ or coinfection with simian immunodeficiency virus (SIV), as seen in TB-human immunodeficiency virus (HIV) coinfections in humans^8,14,15^. As such, cynomolgus macaques have higher potential as animal models than rhesus macaques in TB pathogenesis fields^8,9,16^.

Due to the latent, subclinical and reactivated period, the infected cynomolgus macaques have a high risk of spreading TB to others in the colonies and retransmitting TB back to humans, emphasizing the risk of free-range cynomolgus macaques living in proximity to humans, particularly those kept in sanctuaries, zoo, or research centers. Thus, early TB detection in monkey populations could decrease the spread of the disease among humans, monkeys, and other TB-susceptible wildlife. Generally, there are two practical methods used for TB diagnosis in NHPs and humans: microbiological methods, such as TB culture and acid-fast Bacilli (AFB) staining, and cellular immunological tests, such as the tuberculin skin test (TST) and interferon gamma (IFN- γ) release assay (IGRA)^11,17^. However, there is no “gold-standard” method the diagnosis of latent TB infection.

The TST, which involves intradermal injection of Mammalian Old Tuberculin into the eyelid or abdominal skin, has been routinely used as the standard TB screening tool in NHPs^11,17,18^. This test, however, has several limitations in terms of specificity, sensitivity, time consumption, and reliability^4,11,19–21^. Therefore, new additional tests are required to complement the TST. In the past, two commercial TB diagnostic kits, PrimaGAM (Prionics USA Inc., La Vista, Nebraska, USA) and PrimaTB STAT-Pak (Chembio Diagnostic Systems, Inc., Medford, New York, USA), were available for TB diagnosis in NHPs^17,21^. However, for business reasons, those products have been discontinued.

In humans, two IFN-γ release assays (IGRAs) were approved by the U.S. Food and Drug Administration (FDA) to aid the diagnosis of *M. tb* infection. These tests are the QuantiFERON-TB Gold In-Tube test (QFT-GIT) and the T-SPOT.TB test (T-Spot). The principles of these two assays are similar; the white blood cells from TB-infected hosts are exposed to specific *M. tb* antigens (ESAT6 and CFP10), and the released IFN-γ is measured. Thus, both latent and active infection could be detected^22,23^. Previously, QFT-GIT had been used to detect *M. tb* infection in chacma baboons (*Papio ursinus*)^4^ and *M. kansasii* infection in rhesus monkeys^3^. Recently, the 4th generation of QuantiFERON-TB Gold-Plus (QFT-Plus) (QIAGEN, USA), with improvements in sensitivity for the detection of latent TB infection (LTBI), has been introduced and replaced the former QFT-GIT^23^.

Thailand, located in the center of Southeast Asia, is among the highest-burden TB countries worldwide, with high TB incidences and increasing reports of multidrug-resistant TB (MDR-TB)^24^. In Thailand, cynomolgus macaques commonly inhabit human settlements, temples, and tourist attraction sites. These extensive human-monkey interactions can potentially promote cross-species transmission of the disease^25^. To date, in Thailand, no studies have been conducted on this issue. In this study, we established the mIGRA, which is a combination of the human QFT-Plus and the monkey IFN-γ ELISA^pro^ systems, and used it to investigate TB infection in a group of 39 cynomolgus monkeys at Krabok-Koo Wildlife Breeding Center. These monkeys were cage-mates or lived in cages located near that of a monkey naturally infected with TB that died a month before the onset of the study. The performance of the mIGRA was compared with those of the TST and TB culture.

## Results

### Validation and Interpretation of IFN-γ values

First, white blood cells from the whole-blood samples of monkeys were stimulated in the four format tubes of the human QFT-Plus kit, leading to the release of monkey IFN-γ. The levels of IFN-γ were determined by using a human IFN-γ ELISA kit. However, with this process, the monkey IFN-γ concentrations obtained from four QFT-Plus tubes, including the mitogen (MIT) tube, were invalid. A commercial monkey IFN-γ ELISA^pro^ kit was then used instead of the human IFN-γ ELISA kit, and the monkey plasma IFN-γ levels were successfully determined with the optimal plasma dilution at 1:4. These results indicated that the monoclonal anti-human IFN-γ antibody provided in the human IFN-γ ELISA kit was highly specific for human IFN-γ and did not cross-react with that of cynomolgus monkeys. Since the four format tubes (Nil, TB1, TB2 and MIT) of the human QFT-Plus kit were used for the stimulation of monkey white blood cells to release IFN-γ and the monkey IFN-γ ELISA^pro^ kit was used to determine the monkey IFN-γ concentrations, the test was hereafter named monkey-IGRA (mIGRA).

**Figure 1:**
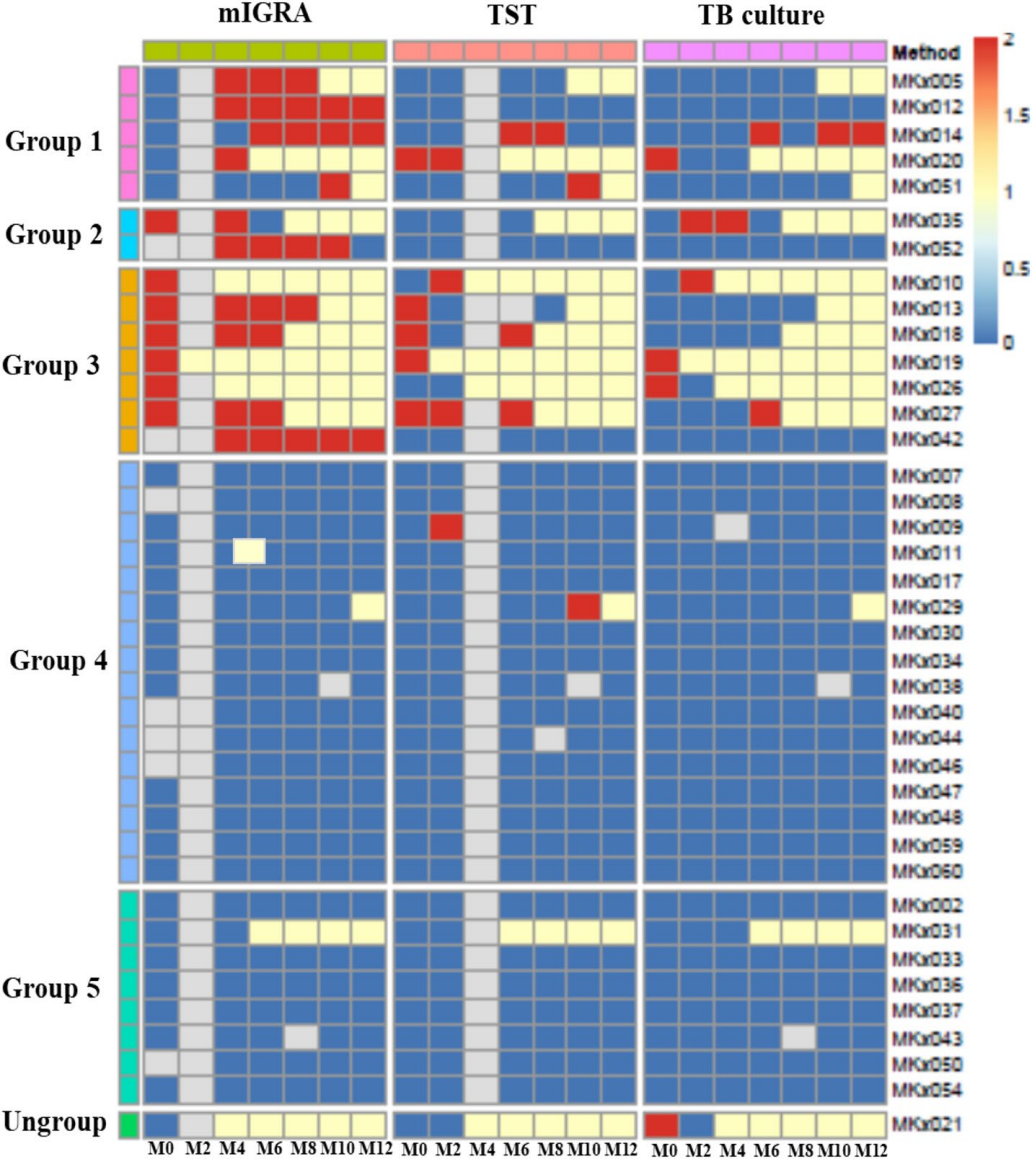
Monkey groups among 39 cynomolgus monkeys**. **The monkeys were categorized on the basis of the results of the three TB diagnosis methods (mIGRA, TST, and TB culture) according to time (months, M). For M0, M2, M4, M6, M8, M10 and M12, a number means a record was present for that month. All results are presented as a heat map (please see Table S3 for more details). Positive-mIGRA, positive-TST, suspected-TST and positive-TB culture are scored as 2 (red), while indeterminate-mIGRA, negative-mIGRA, negative-TST and negative-TB culture are scored as 0 (blue). The heat map was created by Rstudio Version 1.3.1073 at https://www.rstudio.com (pheatmap package).

For analysis, TB infection was first interpreted by Eqs. 1–3 (see “Methods” and Tables S2) and compared with two other TB screening methods (TST and TB culture) (Fig. 1, Tables 1, and S3). It was shown that the mIGRA results gave fewer indeterminate (ID) and incongruent results after evaluation with Eq. 2 compared with the TST and TB culture results. An example of agreement between Eq. 2 and the TST and/or TB culture method was found in Monkey No. MKx018. At month 0, her TST result was positive and her mIGRA results were negative, positive, and indeterminate after evaluation by Eqs. 1–3, respectively (Tables S2 and S3). When retested at months 4 and 6, MKx018 showed positive results evaluated by Eq. 2 until death. Another example was Monkey No. MKx020 (Table S2). At month 4 in MKx020, the mIGRA result was interpreted to be positive by Eqs. 1 and 2 but indeterminate by Eq. 3, and the monkey died suddenly during blood collection. Necropsy examination confirmed that MKx020 was infected with TB, and this result was supported by a positive TB culture. Henceforth, the IFN-γ concentrations were calculated and interpreted using Eq. 2.

**Table 1:** Comparison among three TB tests (mIGRA, TST, and TB culture) in 39 cynomolgus monkeys (**A**) Agreement between the mIGRA and the TST, (**B**) Agreement between TB culture and the mIGRA and between TB culture and the TST.

TST	mIGRA					
	Negative	Positive	*Total*			
Negative	23	6	*29*			
Positive	2	8	*10*			
*Total*	*25*	*14*	*39*			
**Cohen’s kappa coefficient (κ) value**	0.524	Moderate agreement				
***p*-value**	0.0003					
**95% Confidence interval (CI)**	0.2434–0.8053					

TB culture	mIGRA			TST		
	Negative	Positive	*Total*	Negative	Positive	*Total*
Negative	24	7	*31*	26	5	*31*
Positive	1	7	*8*	3	5	*8*
*Total*	*25*	*14*	*39*	*29*	*10*	*39*
**Cohen's kappa coefficient (κ) value**	0.508	Moderate agreement		0.424	Moderate agreement	
***p*-value**	0.0004			0.0120		
**95% Confidence interval (CI)**	0.2290–0.7868			0.0932–0.7555		

Cohen's kappa coefficient (κ) values,* p*-values and 95% confidence intervals (CIs) were calculated by Rstudio Version 1.3.1073 at https://www.rstudio.com (vcd package). The κ-value in the range of 0.41–0.6 means moderate agreement^26^. The italics indicate a total number of monkey cases found positive or negative. Bold is used for topics only such as Kappa value, *p*-value and CIs.

### Determination of TB infection in cynomolgus monkeys based on mIGRA results

We can categorize 39 monkeys in this study into five groups based on the mIGRA results (Fig. 1 and Table S3). In Group 1, the mIGRA results turned from either *negative* or *indeterminate* at month 0 to *positive* after months 4, 6, or 10 (Monkey Nos. MKx005, MKx012, MKx014, MKx020, and MKx051). Three monkeys in this group (Monkey Nos. MKx005, MKx020, and MKx051) ultimately died. Interestingly, MKx014 was confirmed to be TB-positive by the culture test at month 6. At that time, the mIGRA and TST results also turned from negative to positive. However, the TST result changed back to be negative at month 10 and month 12, although a positive TB culture was detected and the mIGRA result remained positive. In Group 2, the mIGRA results turned from *positive* to *indeterminate or negative* for 2 monkeys (Monkey Nos. MKx035 and MKx052). The mIGRA results indicated that Monkey No. MKx035 was infected with TB at months 0 and 4, but the assay result changed to *indeterminate* at month 6 prior to the animal’s death. Notably, the TB culture result was positive at month 4. For Monkey No. MKx052, the mIGRA results were positive at months 4, 6, 8, and 10 until the assay result changed to negative again at month 12. However, the results of the TST and TB culture suggested that this monkey was TB-negative. In Group 3, the mIGRA results remained positive throughout the study period (Monkey Nos. MKx010, MKx013, MKx018, MKx019, MKx026, MKx027, and MKx042). Hence, the mIGRA results for Group 3 could reflect the process of TB development. Additionally, the mIGRA revealed positive results earlier than the TB culture test. In addition, once monkeys were detected to be positive for TB with the mIGRA, the animals remained mIGRA-positive for a long period of time until they tested positive in TB culture and ultimately died. In this group, Monkey No. MKx042 was still alive with prolonged mIGRA-positive results but remained TB-culture-negative to the end of this study. In Group 4, the mIGRA results switched between *negative* and *indeterminate*, whereas other tests showed negative results (Monkey Nos. MKx007, MKx008, MKx011, MKx017, MKx030, MKx034, MKx038, MKx040, MKx044, MKx046, MKx047, MKx048, MKx059, and MKx060), except Monkey No. MKx009 at month 2 and Monkey No. MKx029 at month 10, which showed TST-positive results (Fig. 1). In Group 5, the mIGRA results were shown to be *negative* throughout the 12-month period by all three tests (Monkey Nos. MKx002, MKx031, MKx033, MKx036, MKx037, MKx043, MKx050, and MKx054). Interestingly, Monkey No. MKx031 was the only monkey with negative results in both the mIGRA and culture tests but died later in month 6. Unfortunately, its cause of death was not investigated. Finally, Monkey no. MKx021 was unable to be categorized into any group because his mIGRA result was evaluated as *indeterminate* at month 0, whereas his TB culture test was positive, and he died a month later.

### Comparison among the mIGRA, TST, and TB culture methods

**Figure 2:**
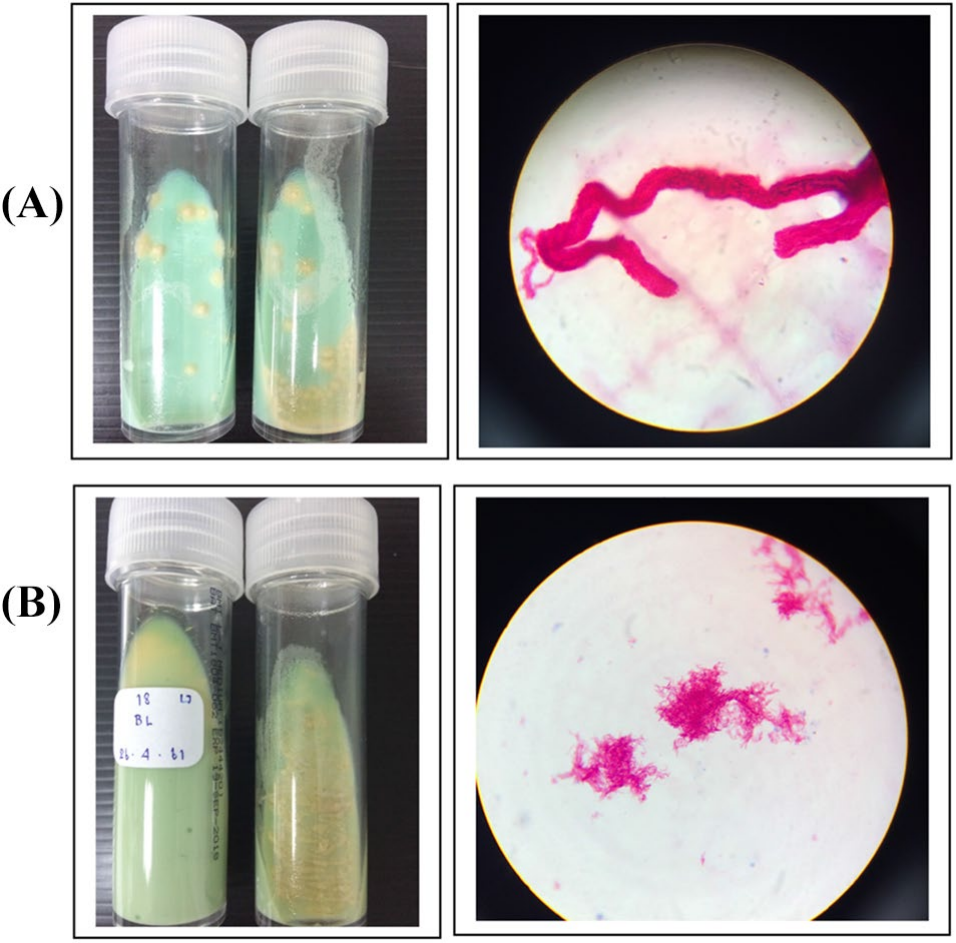
Representative images of colony morphology and AFB staining from two TB-positive cultures (**A**, **B)**. TB colonies (cream color) grown on Lowenstein-Jensen (LJ) media at 37 °C for 2 weeks (left panel) and AFB staining as viewed under a microscope (1000×) (right panel).

Representative images of TB-positive culture, later confirmed by the multiplex PCR method and AFB staining, are shown in Fig. 2. Comparisons among the mIGRA, the TST, and TB culture are presented in Fig. 1 and Table 1. In cynomolgus monkeys, TB-positive results were found in 35.9% (14/39), 25.6% (10/39), and 20.5% (8/39) by the mIGRA, the TST, and TB culture, respectively. Unexpectedly, up to 74.4% (29/39) of the TB-negative results were from the TST. Moderate agreements of the TB test results between two of the three methods are reported: between the mIGRA and the TST (kappa = 0.524; *p*-value 0.0003) (Table 1A), between the mIGRA and TB culture (kappa = 0.508; *p*-value 0.0004) (Table 1B), and between the TST and TB culture (kappa = 0.424; *p*-value 0.012) (Table 1B).

In this study, postmortem examination could be performed in two cynomolgus monkeys (Monkey No. MKx020 and MKx026) because they died during the sample collection, while in 6 other dead monkeys (Monkey No. MKx005, MKx013, MKx018, MKx029, MKx031 and MKx051) with negative culture results, necropsy could not be performed because their carcasses were disposed before we were informed of their death.

Eight *M. tb*-infected monkeys should be given particular attention with regard to their TB-culture-positive results (Tables 1B, 2, S3 and S4). Positive results provided by all three methods (mIGRA, TST, and TB culture) were found in only 5 out of 8 monkeys (62.5%; Monkey Nos. MKx010, MKx014, MKx019, MKx020, and MKx027). Of the other 3 culture-positive cases, Monkey No. MKx026 had positive mIGRA result, Monkey No. MKx021 had indeterminate mIGRA result and Monkey No. MKx035 had positive mIGRA results twice before having indeterminate mIGRA results before death. Although Monkey MKx035 had an indeterminate result with the mIGRA at month 6 prior to its death, it already showed mIGRA-positive results at months 0 and 4 with positive culture. This finding could demonstrate that at month 6, Monkey No. MKx035 had a defect in the cell-mediated immune system, resulting in a change in mIGRA results. More evidence is required. With the TST method, 3 monkeys (Monkey Nos. MKx021, MKx026, and MKx035) exhibited negative results. The results of Monkey No. MKx021 were questionable, as the TB culture showed a positive result, whereas the mIGRA method showed an indeterminate result and the TST result was negative. Taken together, these results indicate that once monkeys were determined to be TB-positive by the TB culture and TST methods, they were absolutely (100%) positive by the mIGRA method (Table 2, mIGRA column). On the other hand, if the mIGRA showed positive results (Table 2, mIGRA row), the monkeys were determined as positive by the TST and TB culture methods in only 57.1% and 50% of the samples, respectively. This finding clearly indicates that the mIGRA is the most sensitive test method among the three methods investigated.

**Table 2:** Percentage of monkeys showing positive mIGRA results compared with the TST and TB culture methods.

TB test	mIGRA	TST	TB culture
mIGRA (n = 14)^a^	100% (14/14)^b^	57.1% (8/14)^b^	50% (7/14)^b^
TST (n = 8)^a^	100% (8/8)^b^	100% (8/8)^b^	62.5% (5/8)^b^
TB culture (n = 7)^a^	100% (7/7)^b^	71.4% (5/7)^b^	100% (7/7)^b^

The numbers in parentheses indicate the number of monkeys.

^a^Indicates numbers of monkeys showing positive results with that method.

^b^Indicates numbers of positive monkeys compared between the two methods; the left number is for the method used in that column, and the right number is for the method used in that row. For example, by comparing the mIGRA and TST methods in row 1, columns 2 and 3, 14 monkeys had positive results with the mIGRA, but only 8 and 7 monkeys were shown to be positive by the TST and TB culture, respectively.

**Figure 3:**
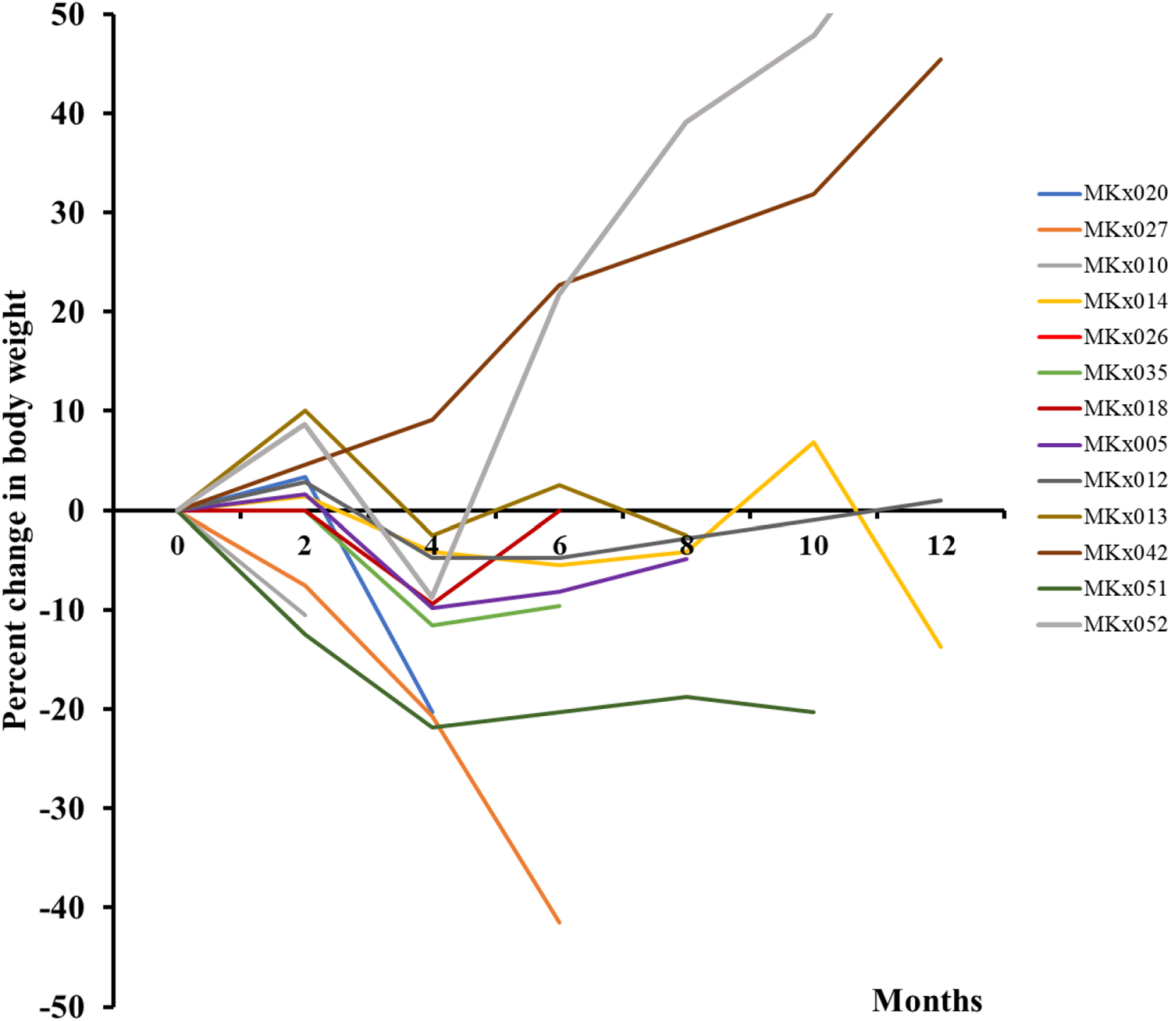
Percent changes in body weight (kilograms; kg) over time (months) in 13 mIGRA-positive monkeys. More details are shown in Table S5.

In addition to the three diagnostic tools mentioned above, it was observed that the body weights of the 5 monkeys (Monkey Nos. MKx010, MKx014, MKx020, MKx027, and MKx035) that showed positive mIGRA and TB culture results obviously declined (10–42% weight loss from month 0) throughout the investigation period (Fig. 3 and Table S5). Thus, the continuous decrease in body weight might be used as a simple physical indicator to assist in the detection of TB in cynomolgus monkeys.

## Discussion

As long as NHPs are used as experimental animal models for the assessment of the safety and efficacy of drugs and vaccines before human use or kept in zoological gardens, strict annual health checks or quarantine programs are needed^8,9,27^. With high demands, primate research facilities in the United States of America (USA) and Europe have to import cynomolgus macaques from the Philippines, Indonesia, Malaysia, and China. Required by regulation from the Centers for Disease Control and Prevention (CDC), these imported monkeys must be TB-free. Unfortunately, TB outbreaks are still present in imported macaques after the quarantine period, leading to research disruption and high expenses associated with disease control^2^*.* One of the reasons for undetectable TB during the quarantine period is that cynomolgus macaques can have LTBI, therefore, a false-negative result can be acquired^7,21^. In addition, animals in which TB is active may show no overt signs of the disease for weeks or months, during which time they can transmit the disease to others in the colony and probably retransmit the disease to humans^19^. Therefore, a highly sensitive, robust, and effective tool to monitor TB infection is necessary, especially at an early stage.

The TST is commonly used for TB diagnosis in many NHP facilities, and the occurrence of false-negative and reactivation cases from latent infection that was undetectable by the TST method is of serious concern^1,17^. Moreover, the TST is not practical for large-scale screening in group-housing systems. Additionally, the periodic unavailability of mammalian old tuberculin has driven recent efforts to develop and validate additional tools for TB diagnosis^21^. Hence, the IGRA has emerged as an earlier powerful technique to detect early TB infection based on the cell-mediated immunity principle^1,20^.

Previously, the human QFT-GIT kit was modified to examine *M. tb*-infected baboons and *M. kansasii*-infected rhesus macaques in the laboratory^3,4^. However, it has never been tested in cynomolgus monkeys that were naturally infected with TB. Apart from the QFT kit, an IFN-γ enzyme-linked immunosorbent spot (ELISPOT) assay is another tool popularly used for TB diagnosis in NHPs under the same principle^8,9,11^. The disadvantages of ELISPOT are the requirement of a large volume of blood and the isolation of peripheral blood mononuclear cells (PBMCs) before the assay^28^. Herein, we introduced the whole-blood mIGRA, which was a combination of the two assay systems (human QuantiFERON-TB Gold-Plus and monkey IFN-γ ELISA^pro^ kits), and we compared its results with those of the well-established methods, the TST and TB culture. The animal subjects recruited for this study were cynomolgus macaques that were naturally infected with TB in wild populations, which could differ from the *M. tb* laboratory strain used.

To interpret TB infection from IFN-γ values of the mIGRA, three equations were applied and revealed that the interpretation by Eq. 1 (QFT) and Eq. 3 might not be suitable because of the equivocal cutoff value based on human data and the ignorance of the NIL outlier values. By Eq. 2, all monkey IFN-γ values of NIL tubes were used to define the cutoff value as well as other criteria as indicated in the “Methods”. As such, the cutoff value is important for TB diagnosis in cynomolgus monkeys and needs to be properly clarified. More data from a large cohort study are also required. Nevertheless, the mIGRA method was shown to be a quick surrogate tool in detecting naturally TB-infected monkeys, covering all three stages of TB infections: asymptomatic or latent, subclinical, and active. This mIGRA should aid TB screening programs for TB management in primate research units. The progression of TB infection from asymptomatic or latent to active could possibly be described. For example, MKx014 was initially determined to be TB-negative by all three tests. However, the monkey was confirmed to be TB-positive by all three methods (mIGRA, TST and TB culture) at month 6, suggesting that it possibly showed signs of being infected before month 6. Later, its TST results became negative, whereas mIGRA and TB culture results were positive. Another example was MKx027, which showed a latent period from month 0 to 4 before reactivation to active infection at month 6 and death. Using the mIGRA, the monkey showed a positive result at months 0 to 4, while the culture result was negative. At month 6, the TB culture result turned positive, while the mIGRA results were constantly positive, and the monkey ultimately died. However, the mIGRA showed a limitation of detection; for example, monkey MKx052 was shown to be mIGRA-negative at months 0 and 12, but it was recorded as mIGRA-positive at months 4, 6, 8, and 10. This finding may indicate that the animal was indeed exposed and infected before month 4, but its CMI-responding cells at month 12 were probably defective in the release of IFN-γ after stimulation by the specific TB antigens coating in TB1 and TB2 tubes, although the IFN-γ level in the MIT tube was normal. More examinations, such as lymphocyte count and thoracic radiography, should be applied as additional tools, and several factors, such as host immunity, technical errors, and coinfection, should be considered^1,17,29–32^.

More importantly, 14 out of 39 monkeys with mIGRA-positive results could be identified as having either LTBI or active TB infection. Later, 7 out of these 14 mIGRA-positive monkeys were confirmed to have subclinical or active-stage infection by the TB culture method. The remaining seven monkeys in this group were determined to have LTBI because of the negative TB culture result. Hence, attention should be paid when monkeys show positive results with mIGRA and TST methods, but negative with TB culture; a culture-negative result may not always mean TB-free, and both false-positive and false-negative results might be produced by the mIGRA and TST methods^1,7,9,17,22,31,32^. As shown in the results, only moderate agreements were achieved when pair comparisons were made among the mIGRA, TST and culture methods, demonstrating that no method should be used alone.

Interestingly, only 8 out of 14 mIGRA-positive monkeys tested positive by the TST, indicating that the TST method is not as sensitive for TB detection as the mIGRA method. In addition, the TST should be performed by a clinical professional because this method is highly subjective. This view is supported by 37.5% of TST-negative results being later found as *positive* by TB culture. Taken together, these results suggest a very high risk for TB outbreaks if TST is used as the sole method in TB screening for cynomolgus monkeys. In addition, TST and mIGRA methods gave conflicting results in 6 monkeys (TST-negative, but mIGRA-positive). Although these two methods use the same principal concept based on T cell response, with the TST being the in vivo test and the mIGRA being the in vitro test, a difference in result outcomes could be found. This showed that, as a benefit, the mIGRA (the IFN-γ test) showed an earlier immune response and was more sensitive than the TST for TB detection in cynomolgus monkeys, with similar sensitivity to that found in cattle with confirmed *M. bovis* infection^20^. This may be explained by the fact that the TST is a delayed-type hypersensitivity (DTH) response measuring both effector and memory T cell responses, whereas the mIGRA mostly measures effector T cell responses. In the in vitro mIGRA system, after 20 h of incubation, circulating effector memory T cells have sufficient time to produce IFN-γ, while the TST might require enough time for central memory T cells, macrophages, and other leukocytes to infiltrate the site of antigen exposure and release cytokines, including IFN-γ, causing induration at the intradermally injected site after 24, 48 and 72 h^33^. Although TB culture is a supportive method for TB diagnosis, this method requires intensive skill, special specimen handling in a Biosafety Level 3 laboratory and an extensive amount of time (1–2 months). Compared to TB culture, the mIGRA is less time-consuming; thus, TB outbreaks can be detected immediately, and preventive measures quickly designed. However, it must be kept in mind that the QFT-Plus kit might give a positive result when animals are infected with *M. kansasii*, *M. szulgai* or *M. marinum*^22^. In addition, a gold-standard method to determine LTBI has not yet been implemented. It is suggested that a diagnosis of *M. tb* infection should include epidemiological and historical data.

**Figure 4:**
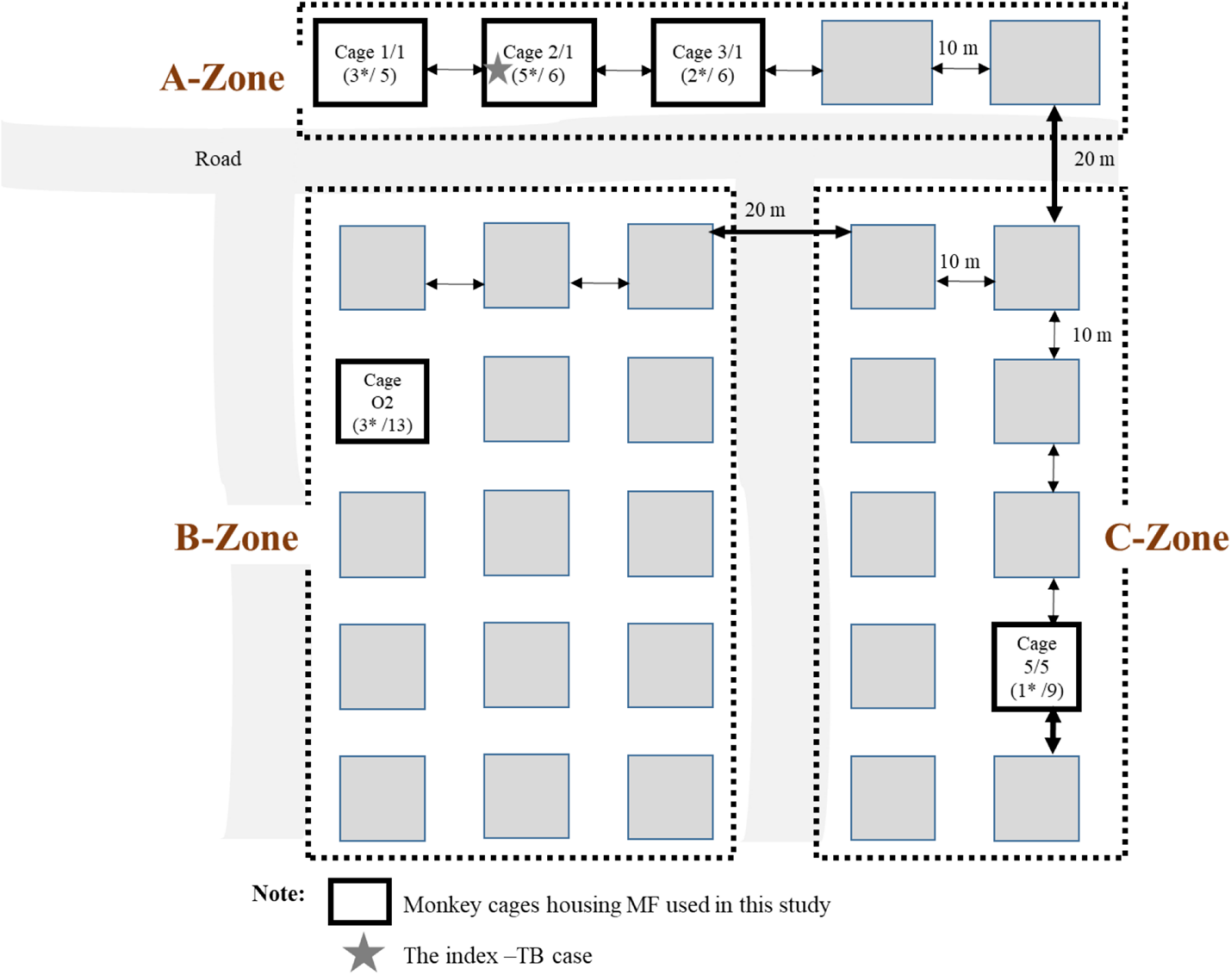
Map drawing of selected monkey cages *(*a box with solid line*)* for the study at Krabok-Koo Wildlife Breeding Center, Tha Takiap District, Chachoengsao Province. The number in brackets indicates the total number of monkeys housed in the cage, and the asterisk (*) indicates the number of mIGRA-positive monkeys. Details of monkeys in these 5 selected cages are summarized in Table S6. The star indicates the index-TB case.

Concerning TB transmission between cages, in this study, it could be as far as 50 m in the open housing system of cynomolgus macaque enclosures (Fig. 4, Table S6). Here, the TB outbreak might have originated from a monkey housed in cage 2/1 (the index TB-cage), and thus, all cage-mates died successively in chronological order. The other mIGRA-positive cases were also found in the nearby cages in the same A-zone, located 10 m apart (cages 1/1 [60%] and 3/1 [33.3%]). In the other two cages (cage O2 in the B-zone and 5/5 in the C-zone), the TB-positive incidence was lower, at 23% and 11%, respectively. With this scenario, TB transmission probably started from direct contact between animals and/or humans (animal caretakers), mainly via aerosol inhalation^6,34–36^. Studies of TB transmission have revealed that such aerosols can be transmitted over short and long distances. In a closed-building area, short-range transmission occurs over a distance of less than a meter between individuals. Long-range transmission occurs between distant locations and is primarily governed by air flows^36^. Moreover, other factors might play roles, such as TB strain (virulence level), meteorological factors (e.g., temperature, humidity, wind, and sunshine), and host factors (immunity and host susceptibility)^36,37^. To have a better explanation of TB transmission, an epidemiological study by phylogenetic analysis is required and should be explored in both animals and human workers. An important lesson learned from this study is that whenever a TB outbreak occurs in NHPs housed in an open system, several preventive measures should be immediately planned and acted upon.

As a reminder, the infection and development of *M. tb* might vary across different species of NHPs^9^. Thus, the mIGRA might not be applicable for other NHP species, and it needs to be validated before use. Beneficially, the mIGRA is a TB-detection tool that is highly sensitive and effective for use as a high-throughput screening test. Importantly, the mIGRA requires only a single on-site visit, is able to screen for early TB infection in cynomolgus monkeys and covers the full spectrum of TB pathology (subclinical, latent and active stages). Thus, the mIGRA method should be another option as a tool to supplement other existing TB-testing tools in cynomolgus macaques.

## Conclusions

The mIGRA is a new, alternative, supportive blood test for TB diagnosis in cynomolgus monkeys. It offers several advantages over the TST, including high sensitivity, speed, and high throughput potential, and it can be performed in a single on-site visit. The major drawback of this method, however, is the reliability of individual immune status. Neither the mIGRA nor the TST should be used as the sole TB diagnostic tool. To verify TB infection accurately, other methods (such as TB culture or molecular diagnostic tests) and clinical examinations of the animals (such as body weight, chest radiography, and clinical signs) should be taken into consideration.

## Methods

### Animals and housing conditions

Thirty-nine cynomolgus monkeys (29 males and 10 females), aged over 7 years, were subjected to the study. They were housed in gang cages (5–13 animals per cage) exposed to natural environmental conditions at Krabok-Koo Wildlife Breeding Center, Tha Takiap District, Chachoengsao Province, eastern Thailand. There were 3 zones of housing (A, B, and C). The neighboring cages in the same zone were 10 m apart, and different zones were 20 m apart (Fig. 4). The animal subjects either were the cage-mates of the monkey that died from TB (a month prior to this study) (cage 2/1, A-zone) or were housed in the neighboring cages (cages 1/1 and 3/1, A-zone). Two cages (one each) in the B and C zones were randomly selected as representatives of different housing zones. The monkeys were anesthetized with a mixture of Zoletil (3–5 mg per kg) and dexmedetomidine hydrochloride (0.03–0.05 mg per kg) before being subjected to blood and bronchial lavage (BL) sample collections, body weight measurements, the TST, and other physical examinations. The experiment at Krabok-Koo Wildlife Breeding Center was approved by the Department of the National Parks, Wildlife and Plant Conservation, Thailand. The animal handling protocol was approved by the Institutional Animal Care and Use Committee of the National Primate Research Center of Thailand-Chulalongkorn University (NPRCT-CU; Protocol review number: 1423007). All methods were also performed in accordance with the relevant guidelines and regulations.

### Blood collection and plasma IFN-γ determination

Blood samples (4 ml each) were collected by femoral venipuncture from each monkey at 0, 4, 6, 8, 10, and 12 months after the first TB case was informed. In total, there were 184 samples from 39 monkeys. Each blood sample was transferred into a sodium heparinized tube and aliquoted (1 ml each), onsite or within 16 h after collection, into four tubes, labeled NIL, TB1, TB2 and mitogen (MIT), of the QuantiFERON-TB Gold- Plus kit (QFT-Plus; Catalog no. 622536, QIAGEN, USA). The tubes were vertically shaken to suspend the coated antigens in the blood prior to incubation at 37 °C. After a 20-h incubation, the blood sample was centrifuged, and the plasma was separated and stored at − 80 °C until assayed for IFN-γ levels. All steps were performed under biosafety cabinet (BSC) class II (Model NU-440-600E, Nuaire, USA) in the Biosafety Level 3 Laboratory.

To determine IFN-γ levels, a commercial monkey IFN-γ ELISA^pro^ kit (catalog no. M4210M-1HP-10, Mabtech AB, Sweden) replaced the human IFN-γ ELISA kit (QFT-Plus ELISA; Catalog no. 622130, QIAGEN, USA) and was performed as described by the manufacturer’s protocol. The plasma was diluted at a ratio of 1:4 before the test. Prior to the assessment of IFN-γ levels of cynomolgus monkeys, the test should meet two criteria listed in the QuantiFERON-TB Gold-Plus (QFT-Plus) ELISA Package Insert 04/2019. First, the IFN-γ level of the MIT tube must be higher than 12.5 pg/ml. If the level is lower, the result should be interpreted as indeterminate (ID). Second, the IFN-γ level of the NIL tube must be lower than the IFN-γ of the TB1 and TB2 tubes. If the level is higher, the result should be interpreted as indeterminate (ID). After meeting those two criteria, the calculation of the IFN-γ level was performed using three equations. Equation 1 (QFT) followed the manufacturer’s suggestion within the human QFT-plus ELISA kit (QuantiFERON-TB Gold-Plus ELISA Package Insert 04/2019). The result was counted as positive when the TB1–NIL or TB2–NIL values were equal to or higher than 8.25 pg/ml and 25% of the NIL value of each animal. Equation 2 followed that described by Parsons’ team^4^, and the value of the minimal detectable dose (MDD) was calculated as twice the average IFN-γ level of the NIL tubes. If the values of TB1–NIL and/or TB2–NIL of each test were greater than the MDD value, the result was confirmed as TB positive. Equation 3 was as described by Parsons’ group^3^, and the 95th percentile of all NIL values ([IFN-γ]^nil95^) was set as a cutoff value between a nonspecific background and a positive result. Moreover, outlier values of the NIL tube were determined by using box plot analysis, excluded as unusually high [IFN-γ]^nil^ values, and reported as indeterminate. A positive (P) result was inferred when the TB1 or TB2 value was greater than the [IFN-γ]^nil95^ and ([IFN-γ]^TB^ − [IFN-γ]^nil^)/[IFN-γ]^nil^ was over 0.25.

### Bronchial lavage collection and culture

BL was collected using a sterile collection cup with a sterile tube connected to a portable phlegm suction unit (Yuwell 7E-A, China). The samples were treated with NaOH/NALC before being transferred to Lowenstein–Jensen (LJ) slants for inoculation and incubation at 37 °C for 7–14 days or until cauliflower-like and cream-colored mycobacterial colonies were identified and isolated. The clarified TB-positive results from this culture test were confirmed to be MTBC by acid-fast Bacilli (AFB) staining and the one-tube multiplex PCR method^38^. Other interpretations, such as contamination, NTM, and negative results, followed those described by Kent and Kubica^39^. Negative culture was reported when no growth was encountered after 2 months of inoculation. All cultural procedures were handled in the Biosafety Level 3 Laboratory containment at the Department of Microbiology, Faculty of Science, Mahidol University, Thailand.

### Intradermal tuberculosis skin test (TST)

The TST was performed by intradermal injection of 0.1 ml (2500 IU) of Mammalian Old Tuberculin (Aoyama B strain human tuberculin, 100,000 IU/ml, Kaketsuken, Japan) into the edge of the upper eyelid of each monkey. Palpebral reactions were graded with a score of 0–5 at 24, 48, and 72 h (h) after injection. Scores of 1 and 2 indicated negative TB results, and scores of 3, 4 and 5 indicated positive TB results^11,17^. Because the TST score was a subjective judgment that might have a personnel bias, at least two additional TSTs were carried out in at least two-week intervals for confirmation before a final result was determined, and a score of 3 indicated suspected TB in this study.

## Supplementary information

Supplementary Information.
