# Supplementary material for: Detection of tuberculosis in cynomolgus macaques (Macaca fascicularis) using a supplementary Monkey Interferon Gamma Releasing Assay (mIGRA)
Source: Sci Rep. 2020 Oct 7;10:16759. doi: 10.1038/s41598-020-73655-3 (PMC7541520; doi:10.1038/s41598-020-73655-3)
Supplement: Supplementary file 1 — Supplementary Information. [file 41598_2020_73655_MOESM1_ESM.pdf]

Detection of Tuberculosis in cynomolgus macaques (*Macaca fascicularis*) using a supplementary Monkey Interferon Gamma Releasing Assay (mIGRA)

S. Warit[1*], P. Billamas[1], N. Makhao[1], S. Jaitrong[1], T. Juthayothin[1], W. Yindeeyoungyeon[1], K. Dokladda[1], N. Smittipat[1], T. Kemthong[2], S. Meesawat[2,3], N. Kongsombat[2], C. Kraitat[2], T. Prammananan[1], T. Palaga[4], A. Chaiprasert[5] & S. Malaivijitnond[2,3*]

[1] Industrial Tuberculosis Team, Industrial Medical Molecular Biotechnology Research Group, National Center for Genetic Engineering and Biotechnology, National Science and Technology Development Agency, 113 Thailand Science Park, Phahonyothin Road, Khlong Nueng, Khlong Luang, Pathum Thani, 12120, Thailand.

[2] National Primate Research Center of Thailand-Chulalongkorn University, Saraburi, 18110, Thailand.

[3] Department of Biology, Faculty of Science, Chulalongkorn University, Bangkok, 10330, Thailand.

[4] Department of Microbiology, Faculty of Science, Chulalongkorn University, Bangkok, 10330, Thailand.

[5] Office for Research and Development, Faculty of Medicine Siriraj hospital, Mahidol University, Bangkok, 10700, Thailand.

*Co-corresponding authors

Saradee Warit; e-mail: saradee@biotec.or.th

Suchinda Malaivijitnond; e-mail: suchinda.m@chula.ac.th

Table S1 Raw data of IFN-γ values (pg/ml) obtained from NIL, TB1, TB2, and Mitogen (MIT) tubes Monkey plasma samples were diluted to 1:4 and used for determinations of the IFN-γ concentrations with the monkey IFN-γ ELISApro kit (Mabtech AB, Nacka Strand, Sweden). **Equation 2** was used for the calculation and interpretation of TB infection from the mIGRA results (see more details in Methods). There are no mIGRA data for month 2. Gray boxes indicate cynomolgus monkeys whose TB culture showed positive results. P, N, and ID represent positive, negative, or indeterminate mIGRA results, respectively. N/A means not applicable.

sample name	month 0					month 4					month 6				
	NIL	TB1	TB2	MIT	mIGRA Result	NIL	TB1	TB2	MIT	mIGRA Result	NIL	TB1	TB2	MIT	mIGRA Result
MKx019	101.53	677.48	884.23	273.17	P	death									
MKx020	13	17.41	21.86	12.17	ID	27.49	134.7	137.01	87.37	P	death				
MKx027	12.45	112.64	115.52	436.47	P	53.5	1088.28	1012.32	224.38	P	149.85	439.18	466.77	782.57	P
MKx010	14.91	21.36	26.14	35.79	P	death									
MKx014	0	0	0	23.09	N	0	0	4.69	579.33	N	35.65	112.3	106.41	652.85	P
MKx021	0	0	0	5.67	ID	death									
MKx026	47.79	41.55	62	98.9	P	death									
MKx035	98.97	293.48	363.86	113.24	P	211.4	834.84	1020.65	446.53	P	502.5	1070.32	1213.79	287.54	ID
MKx018	0.24	2.36	3.73	21.36	P	14.14	85.92	72.52	87.37	P	188.81	521.74	499.38	149.45	P
MKx005	0	0	0	0.41	ID	0	56.16	60.87	133.49	P	0	45.16	59.59	85.36	P
MKx012	0	0	0	636.13	N	120.61	690	530.33	1908.07	P	0	136.69	118.73	961.21	P
MKx013	0	13	10.89	317.72	P	0	100.92	134.43	131.05	P	0	47.42	55.69	220.59	P
MKx042	N /A					0	291.13	314.22	119.81	P	64.11	626.64	504.93	247.84	P
MKx051	0	0	0	0	ID	0	0	0	645.51	N	0	0	0	520.28	N
MKx052	no blood					0	11.68	23.66	546.93	P	0	12.16	11.94	476.68	P
MKx002	0	0	0	15.71	N	0	0	0	12.59	N	0	0	0	35.35	N
MKx007	0	0	0	9.87	ID	0	0	0	97.5	N	0	0	0	140.38	N
MKx008	N/A					0	0	0	127.02	N	0	0	0	36.26	N
MKx009	0	0	0	92.95	N	0	0	0	1234.41	N	0	0	0	185.62	N
MKx011	0	0	0	48.06	N	0	0	0	41.16	N	0	0	0	222.01	N
MKx017	0	0	0	13.41	N	0	0	0	195.92	N	0	0	0	154.31	N
MKx029	0	0	0	252.09	N	5.34	5.34	11.91	347.82	N	0	0	0	508.45	N
MKx030	0	0	0	140.05	N	0	0	0	31.97	N	0	0	0	18.97	N
MKx031	0	0.71	3.16	28.3	N	0	0	0	886.27	N	death				
MKx033	0	0	0	203.49	N	11.45	14.78	12.82	896.5	N	0	0	0	292.88	N
MKx034	0	0	0	45.25	N	0	0	0	213.8	N	0	0	0	188.67	N
MKx036	0	0	0	97	N	0	0	0	128.23	N	0	0	0	92.25	N
MKx037	0	0	0	137.38	N	0	0	0	367.71	N	0	0	0	244.98	N
MKx038	0	0	0	10.6	ID	0	0	0	154.25	N	0	0	0	54.87	N
MKx040	N/A					0	0	0	37.04	N	0	0	0	206.36	N
MKx043	0	0	0	582.95	N	0	0	0	145.77	N	0	0	0	125.45	N
MKx044	N/A					0	0	0	115.96	N	0	0	0	741.76	N
MKx046	N/A					0	0	0	235.95	N	0	0	0	185.33	N
MKx047	0	0	0	10.6	ID	0	0	0	317.22	N	0	0	0	713.74	N
MKx048	0	0	0	351.9	N	0	0	0	471.9	N	0	0	0	209.56	N
MKx050	N/A					0	0	0	558.28	N	0	0	0	141.05	N
MKx054	0	0	0	495.57	N	0	0	0	856.82	N	0	0	0	901.15	N
MKx059	0	0	0	682.51	N	0	0	0	260.63	N	0	0	0	247.51	N
MKx060	0	0	0	10.31	ID	0	0	0	139.33	N	0	0	0	225.82	N

sample name	month 8					month 10					month 12				
	NIL	TB1	TB2	MIT	mIGRA Result	NIL	TB1	TB2	MIT	mIGRA Result	NIL	TB1	TB2	MIT	mIGRA Result
MKx019	death														
MKx020	death														
MKx027	death														
MKx010	death														
MKx014	23.6	91.9	97.02	139.41	P	80.31	229.94	233.82	428.24	P	206.208	252.768	249.268	373.74	P
MKx021	death														
MKx026	death														
MKx035	death														
MKx018	death														
MKx005	6.89	52.27	56.82	77.9	P	death									
MKx012	26.69	170.33	182.96	1034.67	P	0	71.5	50.73	139.96	P	0	234.424	242.288	2835.116	P
MKx013	51.57	881.82	799.14	698.03	P	death									
MKx042	37.19	719.86	698.62	221.06	P	90.35	664.74	620.06	384.2	P	0	490.008	372.008	212.208	P
MKx051	0	0	0	1137.49	N	0	46	60.46	2513.78	P	death				
MKx052	0	67.92	58.2	776.36	P	0	145.58	168.16	6129.2	P	0	0	0	504.548	N
MKx002	0	2.72	3.5	67.24	N	0	0	0	124.12	N	0	0	0	145.452	N
MKx007	0	0	0	68.47	N	0	0	0	0	ID	0	0	0	259.98	N
MKx008	40.48	39.2	30.75	166.68	ID	7.98	0	0	298.26	ID	0	0	0	304.72	N
MKx009	20.75	2.72	5.15	455.69	ID	0	0	0	698.45	N	0	0	0	1571.244	N
MKx011	69.83	55.17	54.75	123.59	ID	0	0	0	151.42	N	0	0	0	283.6	N
MKx017	5.15	1.88	3.24	272.85	ID	0	0	0	121.19	N	N/A				
MKx029	0	0	0	553.48	N	156.32	0	0	0	ID	death				
MKx030	0.92	0	0	101.75	ID	0	0	0	135.89	N	0	0	0	177.848	N
MKx031	death														
MKx033	0	0	0	206.13	N	0	0	0	1820.14	N	0	0	0	1394.34	N
MKx034	0	0	0	123.15	N	0	0	0	167.37	N	0	0	0	0	ID
MKx036	0	0	0	35.45	N	0	0	0	52.83	N	0	0	0	44.884	N
MKx037	0	0	0	191.43	N	0	0	0	406.82	N	0	0	0	259.612	N
MKx038	0	0	0	0	ID	N/A					0	0	0	71.396	N
MKx040	0	0	0	471.9	N	0	0	0	435.99	N	0	0	0	0	ID
MKx043	N/A					0	0	0	779.97	N	0	0	0	561.568	N
MKx044	28.66	36.47	35.74	580.95	N	0	0	0	0	ID	0	0	0	366.748	N
MKx046	6.04	0.53	1.58	349.12	ID	92.85	0	0	347.33	ID	0	0	0	294.624	N
MKx047	0	0	0	138.67	N	0	0	0	2922.57	N	0	0	0	82.356	N
MKx048	23.6	14.94	14.24	402.08	ID	0	0	0	1635.46	N	0	0	0	619.156	N
MKx050	0	0	0	1047.05	N	0	0	0	2454.27	N	0	0	0	1001.524	N
MKx054	0	0	0	1154.81	N	0	0	0	1946.4	N	0	0	0	2173.944	N
MKx059	3.24	0.53	2.17	549.63	ID	0	0	0	1538.16	N	0	0	0	1144.492	N
MKx060	0	0	0	39.91	N	0	0	0	30.56	N	0	0	0	165.016	N

Table S2 mIGRA results interpreted by using Equation 1, Equation 2 and Equation 3 To determine TB infection from the concentrations of IFN-γ obtained by the mIGRA method, three methods to interpret the results by using Equation 1, Equation 2 and Equation 3 were applied as described in Methods. There are no mIGRA data for month 2. Gray boxes indicate monkeys whose TB culture was positive (culture +). P, N, and ID represent positive, negative, and indeterminate mIGRA results, respectively. N/A means not applicable.

Monkey ID	month 0			month 4			month 6			month 8			month 10			month 12		
	Equation1	Equation2	Equation3	Equation1	Equation2	Equation3	Equation1	Equation2	Equation3	Equation1	Equation2	Equation3	Equation1	Equation2	Equation3	Equation1	Equation2	Equation3
MKx019	P	P ; culture +	ID	death														
MKx020	P	ID : culture +	ID	P	P	ID	death											
MKx027	P	P	ID	P	P	ID	P	P ; culture +	P ; culture +	death								
MKx010	P	P ; culture +	ID	death														
MKx014	N	N	N	N	N	P	P	P ; culture +	ID	P	P	P	P	P ; culture +	ID	ID	P	ID
MKx021	ID	ID : culture +	N	death														
MKx026	P	P ; culture +	ID	death														
MKx035	P	P	ID	P	P	ID	ID	P ; culture +	ID	death								
MKx018	N	P	ID	P	P	ID	P	P	ID	death								
MKx005	ID	ID	N	P	P	P	P	P	P	P	P	P	death					
MKx012	N	N	N	P	P	ID	P	P	P	P	P	P	P	P	P	P	P	P
MKx013	P	P	P	P	P	P	P	P	P	P	P	ID	death					
MKx042	N/A			P	P	P	P	P	ID	P	P	ID	P	P	ID	P	P	P
MKx051	ID	ID	N	N	N	N	N	N	N	N	N	N	P	P	P	death		
MKx052	N/ A			P	P	P	P	P	P	P	P	P	P	P	P	N	N	N
MKx002	N	N	N	N	N	N	N	N	N	N	N	N	N	N	N	N	N	N
MKx007	ID	ID	N	N	N	N	N	N	N	N	N	N	ID	ID	ID	N	N	N
MKx008	N/A			N	N	N	N	N	N	ID	ID	ID	ID	ID	ID	N	N	N
MKx009	N	N	N	N	N	N	N	N	N	ID	ID	ID	N	N	N	N	N	N
MKx011	N	N	N	N	N	N	N	N	N	ID	ID	ID	N	N	N	N	N	N
MKx017	N	N	N	N	N	N	N	N	N	ID	ID	ID	N	N	N	N/ A		
MKx029	N	N	N	N	ID	ID	N	N	N	N	N	N	ID	ID	ID	death		
MKx030	N	N	N	N	N	N	N	N	N	ID	ID	ID	N	N	N	N	N	N
MKx031	N	N	P	N	N	N	death											
MKx033	N	N	N	N	ID	ID	N	N	N	N	N	N	N	N	N	N	N	N
MKx034	N	N	N	N	N	N	N	N	N	N	N	N	N	N	N	ID	ID	ID
MKx036	N	N	N	N	N	N	N	N	N	N	N	N	N	N	N	N	N	N
MKx037	N	N	N	N	N	N	N	N	N	N	N	N	N	N	N	N	N	N
MKx038	ID	N	N	N	N	N	N	N	N	ID	ID	ID	N/A			N	N	N
MKx040	N/A			N	N	N	N	N	N	N	N	N	N	N	N	ID	ID	ID
MKx043	N	N	N	N	N	N	N	N	N	N/A			N	N	N	N	N	N
MKx044	N/A			N	N	N	N	N	N	N	N	N	ID	ID	ID	N	N	N
MKx046	N/A			N	N	N	N	N	N	ID	ID	ID	ID	ID	ID	N	N	N
MKx047	ID	N	N	N	N	N	N	N	N	N	N	N	N	N	N	N	N	N
MKx048	N	N	N	N	N	N	N	N	N	ID	ID	ID	N	N	N	N	N	N
MKx050	no blood			N	N	N	N	N	N	N	N	N	N	N	N	N	N	N
MKx054	N	N	N	N	N	N	N	N	N	N	N	N	N	N	N	N	N	N
MKx059	N	N	N	N	N	N	N	N	N	ID	ID	ID	N	N	N	N	N	N
MKx060	ID	N	N	N	N	N	N	N	N	N	N	N	N	N	N	N	N	N

Table S3 TB diagnosis results of the mIGRA, the TST, and TB culture All results of the mIGRA, the TST and TB culture were obtained from 6-7 rounds of sample collections (2-month time interval). The mIGRA results were reported as positive (P), negative (N) and indeterminate (ID). For the TST, scores of N1+ and N2+ were for negative TB results, and scores of S3+, P4+ and P5+ were for positive TB results. A score of 3 by the TST indicated suspected TB (S3+) in this study. NTM means nontuberculous mycobacteria. Gray boxes indicate monkeys whose TB culture results were positive. N/A means not applicable.

Group	Monkey ID	mIGRA							TST							TB culture						
		month 0	month 2	month 4	month 6	month 8	month 10	month 12	month 0	month 2	month 4	month 6	month 8	month 10	month 12	month 0	month 2	month 4	month 6	month 8	month 10	month 12
group 1	MKx005	ID	N /A	P	P	P	death		N	N	N /A	N	N	death		No growth	NTM	No growth	NTM	contaminated	death	
	MKx012	N	N /A	P	P	P	P	P	N	N	N /A	N	N	N	N	NTM	NTM	contaminated	NTM	contaminated	contaminated	contaminated
	MKx014	N	N /A	N	P	P	P	P	N	N	N /A	P 4+	S 3+	N 1+	N 1+	NTM	NTM	contaminated	*M.tb*	contaminated	*M.tb*	*M.tb*
	MKx020	ID	N /A	P	death				P 4+	S 3+	N /A	death				*M.tb*	contaminated	contaminated	death			
	MKx051	ID	N /A	N	N	N	P	death	N	N 2+	N /A	N	N	S 3+	death	contaminated	NTM	NTM	No growth	NTM	NTM	death
group2	MKx035	P	N /A	P	ID	death			N	N 1+	N /A	N 1+	death			NTM	*M.tb*	*M.tb*	NTM	death		
	MKx052	N/A	N /A	P	P	P	P	N	N	N	N /A	N	N	N	N	NTM	NTM	NTM	NTM	No growth	contaminated	contaminated
group 3	MKx010	P	N /A	death					N	S 3+	death					contaminated	*M.tb*	death				
	MKx013	P	N /A	P	P	P	death		S 3+	N	N /A	N /A	N	death		NTM	NTM	NTM	contaminated	NTM	death	
	MKx018	P	N /A	P	P	death			P 4+	N 2+	N /A	S 3+	death			NTM	contaminated	contaminated	NTM	death		
	MKx019	P	death						P 4+	death						*M.tb*	contaminated	death				
	MKx026	P	N /A	death					N	N	death					*M.tb*	contaminated	death				
	MKx027	P	N /A	P	P	death			P 4+	P 4+	N /A	S 3+	death			contaminate	NTM	contaminated	*M.tb*	death		
	MKx042	N/A	N /A	P	P	P	P	P	N	N	N /A	N	N 2+	N	N	NTM	No growth	contaminated	No growth	NTM	contaminated	NTM
group 4	MKx007	ID	N /A	N	N	N	ID	N	N	N	N /A	N	N	N	N	NTM	NTM	No growth	NTM	contaminated	contaminated	contaminated
	MKx008	N/A	N /A	N	N	ID	ID	N	N	N	N /A	N	N	N	N	contaminated	NTM	No growth	contaminated	contaminated	NTM	contaminated
	MKx009	N	N /A	N	N	ID	N	N	N	S 3+	N /A	N	N	N	N	NTM	contaminated	N / A	contaminated	contaminated	contaminated	contaminated
	MKx011	N	N /A	N	N	ID	N	N	N	N	N /A	N	N	N	N	NTM	contaminated	contaminated	contaminated	NTM	contaminated	contaminated
	MKx017	N	N /A	N	N	ID	N	N /A	N	N	N /A	N 1+	N	N	N	NTM	NTM	No growth	contaminated	contaminated	NTM	NTM
	MKx029	N	N /A	N	N	N	ID	death	N	N	N /A	N	N	S 3+	death	NTM	NTM	NTM	NTM	NTM	NTM	death
	MKx030	N	N /A	N	N	ID	N	N	N	N	N /A	N	N	N	N	NTM	NTM	NTM	contaminated	NTM	contaminated	contaminated
	MKx034	N	N /A	N	N	N	N	ID	N	N	N /A	N	N	N	N	NTM	NTM	NTM	No growth	NTM	NTM	NTM
	MKx038	ID	N /A	N	N	ID	N/A	N	N	N	N /A	N	N	N/A	N	No growth	NTM	NTM	NTM	No growth	N/A	NTM
	MKx040	N/A	N /A	N	N	N	N	ID	N	N	N /A	N	N	N	N	No growth	NTM	NTM	NTM	No growth	contaminated	contaminated
	MKx044	N/A	N /A	N	N	N	ID	N	N	N	N /A	N	N/A	N	N	NTM	No growth	contaminated	NTM	No growth	contaminated	contaminated
	MKx046	N/A	N /A	N	N	ID	ID	N	N	N	N /A	N	N	N	N	NTM	contaminated	NTM	contaminated	No growth	NTM	NTM
	MKx047	ID	N /A	N	N	N	N	N	N	N	N /A	N	N	N	N	NTM	NTM	contaminate	NTM	NTM	NTM	NTM
	MKx048	N	N /A	N	N	ID	N	N	N	N	N /A	N	N	N	N	NTM	contaminated	NTM	contaminated	NTM	contaminated	contaminated
	MKx059	N	N /A	N	N	ID	N	N	N	N	N /A	N	N	N	N	contaminated	NTM	contaminate	No growth	NTM	NTM	NTM
	MKx060	ID	N /A	N	N	N	N	N	N	N	N /A	N	N	N	N	contaminated	NTM	NTM	NTM	NTM	NTM	NTM
group 5	MKx002	N	N /A	N	N	N	N	N	N	N	N /A	N	N	N	N	NTM	No growth	No growth	NTM	contaminated	contaminated	contaminated
	MKx031	N	N /A	N	death				N	N +2	N /A	death				NTM	No growth	No growth	death			
	MKx033	N	N /A	N	N	N	N	N	N	N	N /A	N	N	N	N	NTM	NTM	contaminated	No growth	NTM	contaminated	contaminated
	MKx036	N	N /A	N	N	N	N	N	N	N	N /A	N	N	N	N	contaminated	No growth	NTM	contaminated	NTM	NTM	NTM
	MKx037	N	N /A	N	N	N	N	N	N	N	N /A	N	N	N	N	NTM	NTM	No growth	NTM	NTM	NTM	NTM
	MKx043	N	N /A	N	N	N/A	N	N	N	N	N /A	N	N	N	N	NTM	NTM	NTM	NTM	N/A	contaminated	contaminated
	MKx050	N/A	N /A	N	N	N	N	N	N	N	N /A	N	N	N	N	NTM	NTM	NTM	NTM	NTM	contaminated	contaminated
	MKx054	N	N /A	N	N	N	N	N	N	N	N /A	N	N	N	N	No growth	NTM	NTM	NTM	NTM	NTM	NTM
ungroup	MKx021	ID	N /A	death					N	N	death					*M.tb*	contaminated	death				

Table S4 Summary of TB diagnosis results of 39 cynomolgus monkeys by the mIGRA, TST and TB culture methods These summary results were evaluated from 6-7 rounds of all interpreted results. Gray boxes indicate monkeys whose TB culture results were positive. The mIGRA results were reported as positive (P), negative (N) or indeterminate (ID). For the TST method, the results were reported positive (P), suspected (S), or negative (N). Using the TB culture method, positive (P) and negative (N) results were reported. In this study, an indeterminate mIGRA result was considered negative (N) for TB infection, while a suspected TST (S) result was considered positive for TB infection.

Monkey ID	mIGRA	TST	TB culture
MKx019	P	P	P
MKx020	P	P	P
MKx027	P	P	P
MKx010	P	S (P)	P
MKx014	P	P	P
MKx021	ID (N)	N	P
MKx026	P	N	P
MKx035	P	N	P
MKx018	P	P	N
MKx005	P	N	N
MKx012	P	N	N
MKx013	P	S (P)	N
MKx042	P	N	N
MKx051	P	S (P)	N
MKx052	P	N	N
MKx002	N	N	N
MKx007	N	N	N
MKx008	N	N	N
MKx009	N	S (P)	N
MKx011	N	N	N
MKx017	N	N	N
MKx029	N	S (P)	N
MKx030	N	N	N
MKx031	N	N	N
MKx033	N	N	N
MKx034	N	N	N
MKx036	N	N	N
MKx037	N	N	N
MKx038	N	N	N
MKx040	N	N	N
MKx043	N	N	N
MKx044	N	N	N
MKx046	N	N	N
MKx047	N	N	N
MKx048	N	N	N
MKx050	N	N	N
MKx054	N	N	N
MKx059	N	N	N
MKx060	N	N	N

Table S5 Monkey body weight in kilograms (kg) versus time (months) for 14 positive-mIGRA monkeys The percentage of weight loss (minus value) and weight gain (normal value) compared between month 0 and the final month are presented in the final column. Gray boxes indicate monkeys that had a positive TB culture. N/A means not applicable.

Monkey ID	Body weight (kg) at different time point (months)							Percentage of body weight loss or gain between month 0 and death or the end of the study
	0	2	4	6	8	10	12	
MKx019	2.1	death	death	death	death	death	death	N/A
MKx020	5.9	6.1	4.7	death	death	death	death	-20.34
MKx027	5.3	4.9	4.2	3.1	death	death	death	-41.51
MKx010	3.8	3.4	death	death	death	death	death	-10.53
MKx014	7.3	7.4	7	6.9	7	7.8	6.3	-13.7
MKx026	2.7	2.7	death	death	death	death	death	0
MKx035	5.2	5.2	4.6	4.7	death	death	death	-9.62
MKx018	3.2	3.2	2.9	3.2	death	death	death	0
MKx005	6.1	6.2	5.5	5.6	5.8	death	death	3.28
MKx012	5.25	5.4	5	5	5.1	5.2	5.3	0.95
MKx013	4	4.4	3.9	4.1	3.9	death	death	-2.5
MKx042	2.2	2.3	2.4	2.7	2.8	2.9	3.2	45.45
MKx051	6.4	5.6	5	5.1	5.2	5.1	death	-20.31
MKx052	2.3	2.5	2.1	2.8	3.2	3.4	3.8	65.22

Table S6 Monkey ID number and the number of monkeys (in brackets) from 5 individual cages TB infection results of 39 cynomolgus monkeys in 5 cages (cage nos. 1/1, 2/1, 3/1, O2 and 5/5) are summarized. Numbers (in brackets) indicate the number of monkeys residing in a cage with positive results in the TB diagnostic methods used (mIGRA, TST and TB culture). P, ID, and S mean positive, indeterminate, and suspected, respectively. In this study, ID mIGRA results were considered negative for TB infection, while suspected TST (S) results were considered positive for TB infection.

Cage nos.	Number of monkeys (cases)			
	**Total**	**P or ID - mIGRA**	**P or S -TST**	**P-TB culture**
1/1	MKx014, MKx012, MKx013, MKx011, MKx017	MKx014, MKx012, MKx013,	MKx014 - P, MKx013 - S	MKx014
	(n = 5)	(P = 3)	(P = 1, S = 1)	(P = 1)
2/1	MKx019, MKx020, MKx027, MKx021, MKx018, MKx026	MKx019, MKx020, MKx027, MKx018, MKx026	MKx019, MKx020, MKx027, MKx018	MKx019, MKx020, MKx027, MKx021, MKx026
		MKx021 – ID		
	(n = 6)	(ID = 1, P = 5)	(P = 4)	(P = 5)
3/1	MKx010, MKx005, MKx002, MKx007, MKx008, MKx009	MKx010, MKx005	MKx010 - S, MKx009 - S	MKx010
	(n = 6)	(P = 2)	(S - 2)	(P = 1)
O2	MKx042, MKx051, MKx052, MKx040, MKx043, MKx044, MKx046, MKx047, MKx048, MKx050, MKx054, MKx059, MKx060	MKx042, MKx051, MKx052,	MKx051 – S	undetectable
	(n = 13)	(P = 3)	(S - 1)	
5/5	MKx035, MKx029, MKx030, MKx031, MKx033, MKx034, MKx036, MKx037, MK038	MKx035	MKx029 - S	MKx035
	(n = 9)	(P = 1)	(S = 1)	(P = 1)
